# Calciphylaxis – a challenging & solvable task for plastic surgery? A case report

**DOI:** 10.1186/1471-5945-13-1

**Published:** 2013-01-14

**Authors:** Savas Tsolakidis, Gerrit Grieb, Andrzej Piatkowski, Ziyad Alharbi, Erhan Demir, David Simons, Norbert Pallua

**Affiliations:** 1 Department of Plastic Surgery and Hand Surgery, Burn Center, Medical Faculty, RWTH Aachen University, Pauwelsstrasse 30, 52074, Aachen, Germany

**Keywords:** Calciphylaxis, Phenprocoumon, Natriumthiosulphate, Skin Grafting, Buried Chip skin grafting

## Abstract

**Background:**

Calciphylaxis (calcific uremic arteriolopathy) is rare and its pathogenesis is not fully understood. Indeed, Calciphylaxis presents a challenge through the course of its management which involve different specialities but unfortunately this disease so far has a poor prognosis. We herein present, in this case report, a multidisciplinary approach involving plastic surgeons with special regards to reconstructive approach after debridement procedures.

**Case presentation:**

We present a 21 years old male with a BMI of 38,2, who was transferred to our department from another hospital. Calciphylaxis has been diagnosed after receiving anticoagulation with phenprocoumon after a single event of pulmonary embolism. The INR on admission was 1,79. He had necrotic spots on both sides of the abdominal wall and on both thighs medially. During this time he underwent several reconstructive procedures in our department.

**Conclusion:**

It can be suggested that this agonizing disease needs indeed a multidisciplinary approach involving Nephrologists, Dermatologists, Intensive Care Physicians and Plastic Surgeons, taking into consideration that surgical correction can achieve further improvement in a specialized centre. Notwithstanding, further cohort studies should be approached clinically to insight the light on this disease with special regard to the prognosis after this approach.

## Background

Calciphylaxis (calcific uremic arteriolopathy) is a rare and poorly understood disease which demonstrates a challenge for different specialities. Media calcifications of arterioles and fat tissue are distinctive and primarily affect the skin but in some cases involvement of nerve sheaths, visceral organs and muscles have been described. Even if diagnosed in an early phase, the chances of success in healing are very low and the mortality is exceptionally high [1].

It is characterized by extremely painful, partly necrotic skin ulcerations, which can be found almost on any part of the body. Hallmark of this disease is a fortified calcification of the arterioles mainly in the subcutaneous tissue and sometimes even calcification of peripheral nerve sheaths and fat cell bodies can be found [1]. The primary clinical picture is multifaceted and can delay diagnosis. The differential diagnosis is challenging and includes vasculitis, cholesterol emboli and diabetic ulcers.

Calciphylaxis can be staged into two phases. Phase one is characterized by pruritus, cutaneous laminar erythemas, which is unspecific and therefore can easily be misinterpreted in the first place. Phase two shows painful ulceration and necrosis [2]. Preferably affected areas are the lower extremities. There are some cases described where also the abdomen and visceral organs were affected. The mortality in phase one is about 30%, but the mortality in phase two is extended up to 80% [3]. These typical ulcerations and necrosis were predominantly presented on the abdomen and both thighs of the patient we treated.

This rare disease was first described by Bryant and White in 1898, where a condition of calcifications and cutaneous necrosis was circumscribed [4]. Selye et al. were the first, who highlightened the pathophysiology of calciphylaxis, labelled the disease in 1962 and characterised it as a systemic hypersensitivity reaction. In animal experiments, Selye et al. could show an induction of calcification of various organs via the combination of sensitizing agents, like Dihydrotachysterol, Vitamin D2,D3 and parathyroid hormone followed by exposure to a challenger, as there are for example metallic salts (iron, aluminium)or trauma [5-7]. Ketteler et al. referred the condition can lead to an imbalance of two calcification inhibiting proteins; Matrix-Gla and Fetuin A, which are Vitamin K dependant [8]. In animal experiments it could be shown that an essential lack of these proteins can lead to calcification of arteries and even rupturing of the aorta [9,10].

Even the risk factors seem to be controversial. Predisposing factors are an acute or chronic renal failure, obesity, diabetes, hyperparathyroidism, female Caucasians and an elevated calcium phosphorus product. Further presumably risk factors are phenprocoumon, vitamin K deficiency (phytonadione), lack of fetuin A protein, malignancy, alcoholic liver disease, connective tissue disease, protein C and S deficiency [11]. Interestingly the majority of these patients had normal serum calcium, normal serum phosphorus, normal calcium phosphorus product, normal serum parathyroid hormone levels and normal serum creatinine levels. The mortality rate in this study was 52% with sepsis being the leading cause [12].

Although the risk remains to cause another complicated ulcer, a histological sample excision also including subcutaneous tissue is mandatory. The sample has to be stained with alizarin red or silver-nitrate to differentiate Calciphylaxis from different diseases [11].

Fine et al. suggested bone scintigraphy as one of the first steps to substantiate the diagnosis. In their study, only one of the investigated cases had negative bone scan [13] .The mortality in phase one was 33%. After development of ulcers the mortality reached 80% [13,14].

In this case report, we highlight a multidisciplinary treatment of a patient, suffering from Calciphylaxis, which after 10 months led to a successful result.

### Case presentation

We report on an obese 21 years old male with a Body Mass Index (BMI) of 38,2, who presented to our department with Calciphylaxis after phenprocoumon intake in consequence of pulmonary embolism. The target partial thrombin time (pTT) was between 50-60s. In addition the patient lost overtime 100 kg body mass after getting a gastric banding in 2007. Comorbidities were a dilative cardiomyopathy, insulin dependant diabetes, obesity, allergic asthma, hypothyreoidism and lack of anithrombine factor III (AT3).

Calciphylaxis was already diagnosed histologically in the former hospital, where he was treated with Natriumthiosulphate intravenously for about 9 weeks. Initial symptoms were tender and painful lesions with defined edges in the upper and lower thighs and the abdomen, where some showed signs of necrosis. Further histological examinations showed typical signs of microthrombosis and calcification of the arterioles subcutaneously and substantiated the diagnosis of calciphylaxis.

The patient needed high doses of opiates over the whole duration of inpatient treatment. Unfortunately the initial calcifications became necrotic and possible surgical options were evaluated.

**Figure 1:**
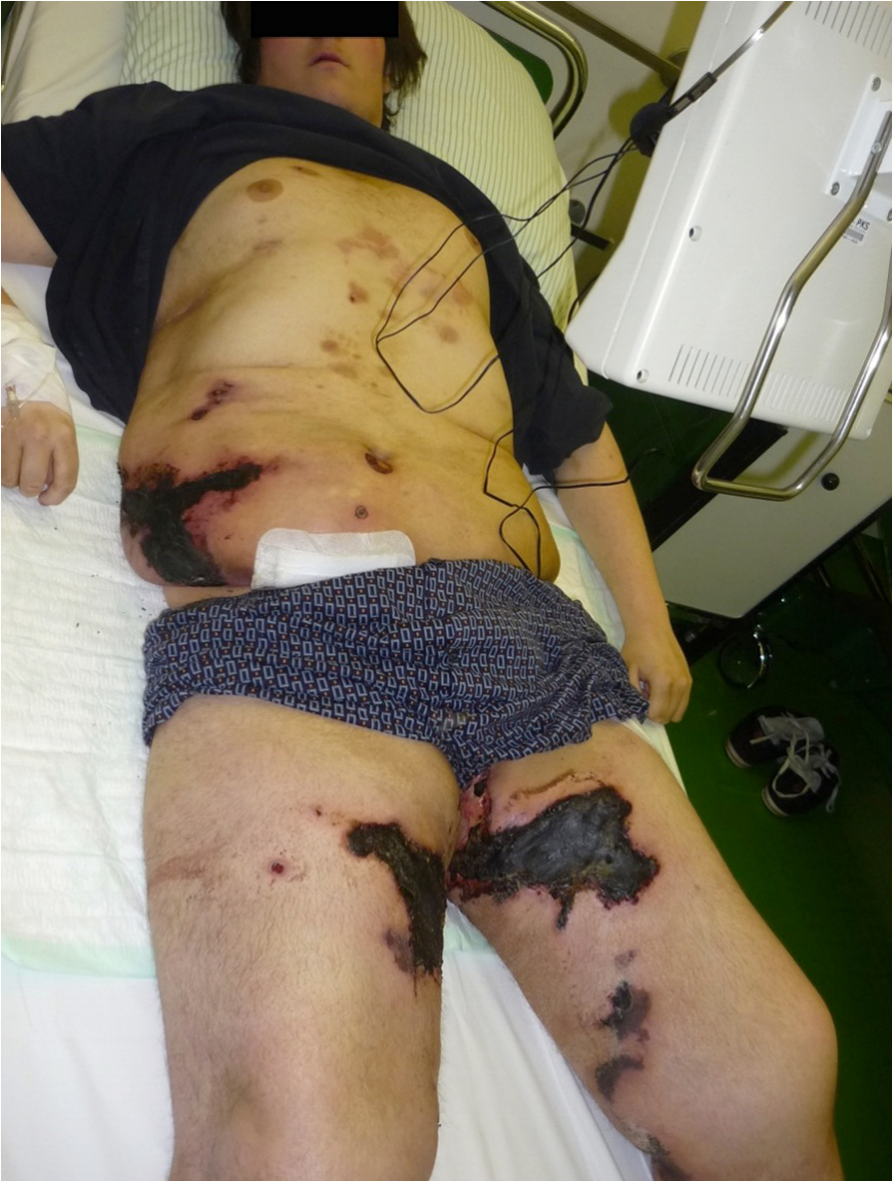
Shows the patient's wounds on both thighs and the abdominal wall on admission in our department.

A combination of Natriumthiosulphate was administered intravenously, which is a common therapy for calciphylaxis and a specific surgical approach to this complex disease was initiated when the patient arrived at our department. The patient had necrosis on the abdomen and both thighs as well as partially superinfected wounds with oxacillin resistant staphylococcus aureus (Figure 1). A bone scintigraphy revealed no increased activity of calcium carbonate in thighs or the abdominal area.

**Figure 2:**
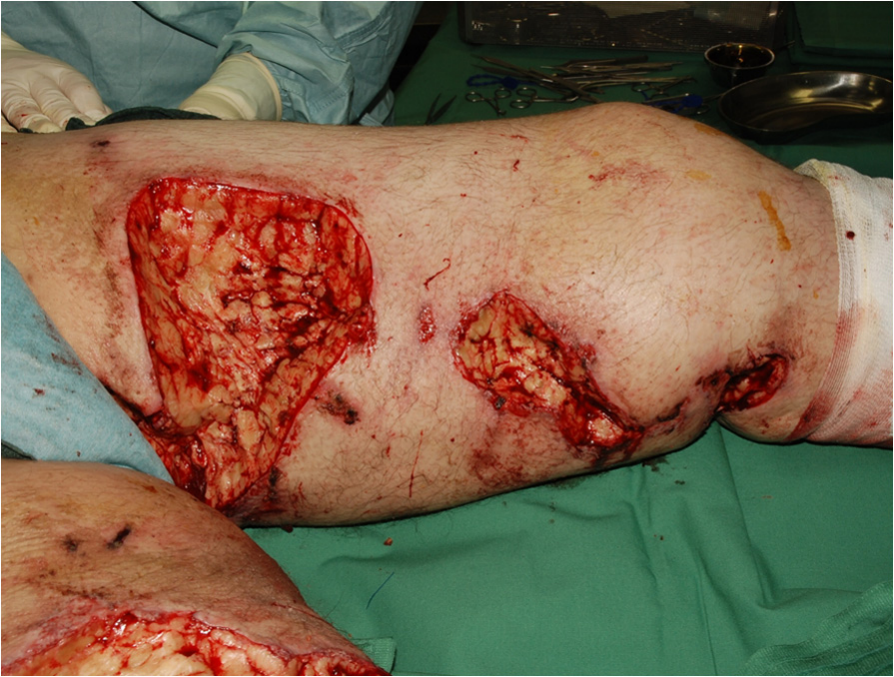
Shows exemplary the wounds on the thigh after initial debridements in our department.

After the patient consented to a surgical treatment we started our surgical approach with extensive debridement of the affected areas on the abdominal wall and both thighs (Figure 2). A consecutive VAC therapy for the lesions on the thighs was mandatory to establish a clean environment. At this level, attempt to close the wounds on the abdomen via advancement flaps were unsuccessful.

A main problem to deal with was not only a permanent level of extensive pain which was encountered through our pain specialists in concordance with the WHO – pain schedule with opiates but also the recurrent wound infections with complicated germs, which were treated with different antibiotic patterns. Over time the Calciphylaxis therapy was adjusted and complemented with Pamidronat, a biphosphonate agent to reduce decomposition of osseous structures and Renagel (Sevelamer), a phosphate binder.

Initial skin grafts did not show satisfactory results in the beginning of the surgical treatment; hence another attempt with skin grafts in buried chips technique was preceded 6 months after admission.

From the plastic surgical point of view, the complexity of the wounds demanded a combination of consecutive debridements, jet lavages, repetitive VAC therapies and Buried chip skin grafting (both thighs and abdomen). In addition dressings were changed with an ointment pad and silver coating dressing (Atrauman Ag, Paul Hartmann Ges.m.b.H, Wiener Neudorf – Austria) as well as antiseptic solution (Polyhexanid, Lohmann & Rauscher GmbH & Co. KG, Neuwied – Germany) on a daily basis. The patient could be sufficiently mobilized and had no further signs of infection. Daily sea salt baths and physiotherapy sessions improved the patient’s status until he was able to walk alone on the ward.

Finally, pain medication could be reduced drastically and sedoanalgesia was not further necessary for dressing changes. The laboratory on discharge showed a single slight elevated phosphate of 1.88 (0.84-1.48 mmol/l). Calcium was in normal range (2.10-2.60 mmol/l). Creatinine was 1.5 (0.7-1.2). We could successfully discharge the patient into rehabilitation, where his status was further improved by mobilization, physiotherapy and lymphatic drainage massages.

## Conclusion

Calciphylaxis still remain a challenge and need further investigation to understand this disease although different causes and numerous therapeutic strategies have been described. A further challenge in the clinical setting is that the therapy for Calciphylaxis is not clearly defined. Independently of the causing agent (factor), the present therapeutic strategy enfolds pain control, surgery, calcium binding agents, phosphorus reducer, cortisone, and antibiotic therapy.

**Figure 3:**
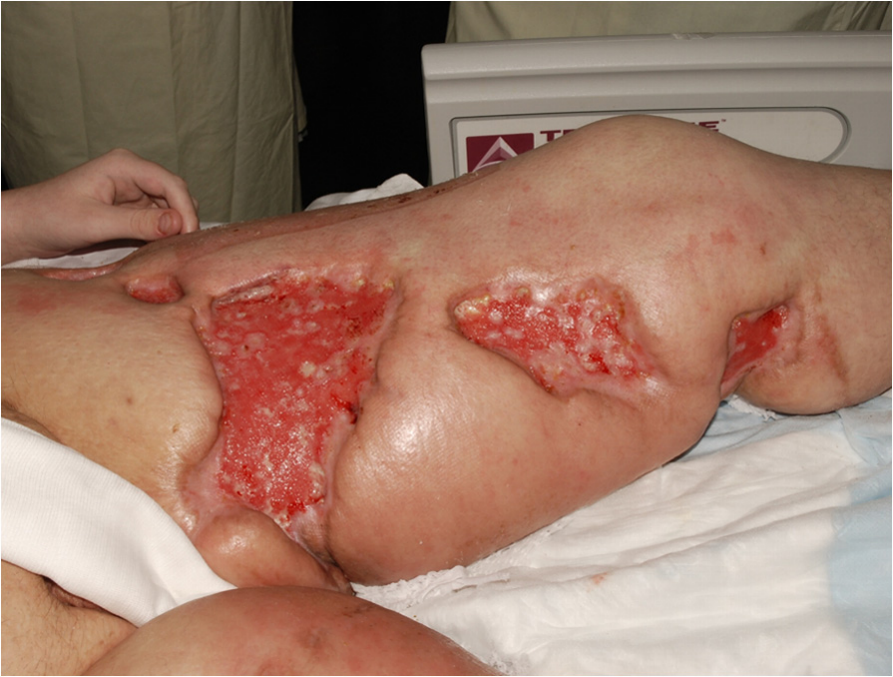
**Shows the clinical setting after successful skin grafting procedures.** The wounds were closed.

Regarding our patient, we treated him with a combination of surgical approach with intravenous application of binding agents which have led to a successful outcome. However, it should be stated that local flap techniques showed no benefit but merely buried skin grafting was finally crowned with success. Therefore an interdisciplinary approach to this disease via supporting drug therapy and surgical measures may be a promising way to treat patients suffering from Calciphylaxis. The patient was then discharged into rehabilitation centre without further wound problems in his soft tissues and until recently showed no signs of a relapse of Calciphylaxis (Figure 3 ).

Notwithstanding, despite the fact that this surgical approach may lead to a satisfactory result in our patient further studies with a larger patient cohort will be needed to determine its efficiency for these patients.

### Consent

Written informed consent was obtained from the patient for publication of this case presentation and any accompanying images.

## Competing interests

The authors have declared that no conflict of interest exists.

## Authors’ contributions

ST drafted the manuscript and participated in the analysis. GG, AP, ZA and DS conceived the design and participated in the treatment and contributed in the revision process. ED treated the Patient in the intensive care unit. NP participated in the operations as well as revised and edited the manuscript. All authors read and approved the final manuscript.

## Pre-publication history

The pre-publication history for this paper can be accessed here:

http://www.biomedcentral.com/1471-5945/13/1/prepub
